# Implementation of neurocritical care in Thailand

**DOI:** 10.3389/fneur.2022.990294

**Published:** 2022-10-18

**Authors:** Tanuwong Viarasilpa

**Affiliations:** Division of Critical Care, Department of Medicine, Siriraj Hospital, Mahidol University, Bangkok, Thailand

**Keywords:** neurocritical care, implementation, Thailand, resource-limited, low and middle-income countries (LMIC)

## Abstract

Dedicated neurointensive care units and neurointensivists are rarely available in Thailand, a developing country, despite the high burden of life-threatening neurologic illness, including strokes, post-cardiac arrest brain injury, status epilepticus, and cerebral edema from various etiologies. Therefore, the implementation of neurocritical care is essential to improve patient outcomes. With the resource-limited circumstances, the integration of neurocritical care service by collaboration between intensivists, neurologists, neurosurgeons, and other multidisciplinary care teams into the current institutional practice to take care of critically-ill neurologic patients is more suitable than building a new neurointensive care unit since this approach can promptly be made without reorganization of the hospital system. Providing neurocritical care knowledge to internal medicine and neurology residents and critical care fellows and developing a research system will lead to sustainable quality improvement in patient care. This review article will describe our current situation and strategies to implement neurocritical care in Thailand.

## Introduction

Neurocritical care integrates critical care and neurological expertise to comprehensively manage patients with life-threatening brain injuries (1, 2). With an advance in evidence-based knowledge and neuromonitoring technology, neurocritical care has become an essential part of the healthcare system in developed countries (3). However, despite a significant disease burden, neurointensive care units (NICU) and neurointensivists are rarely available in Thailand and other low and middle-income countries (2, 4, 5). Accordingly, an effort to deliver neurocritical care is essential to improve patient outcomes in Thailand.

## Current situation of neurocritical care in Thailand

**Figure 1:**
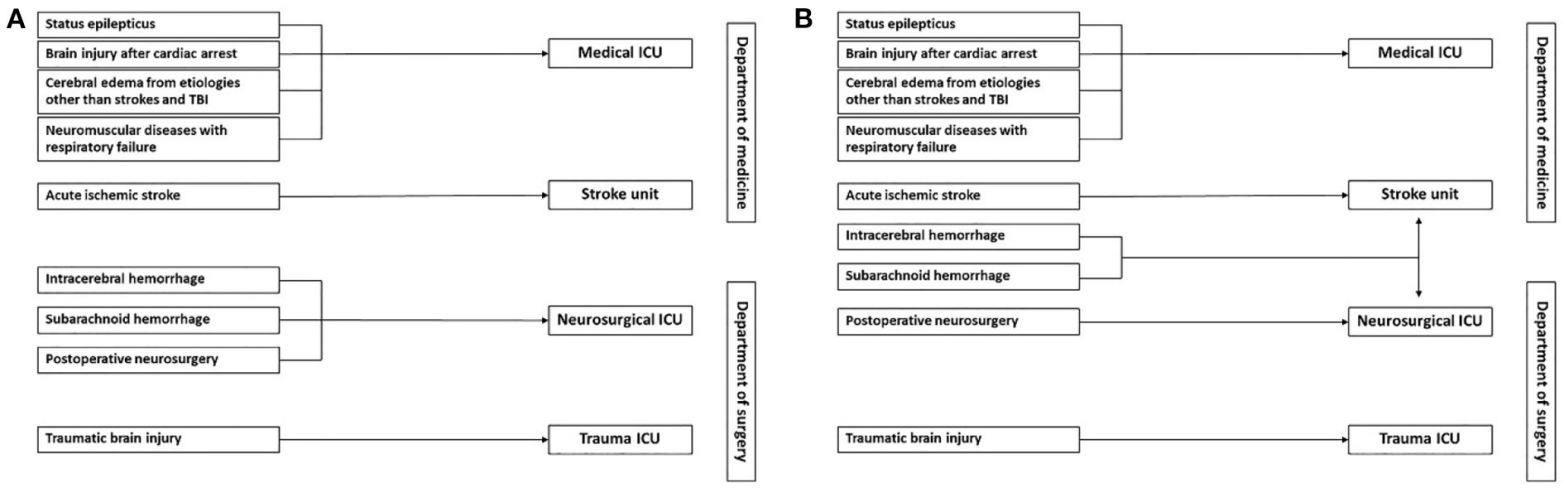
Workflow of neurocritical care patients in our institution before **(A)** and after **(B)** the operation of a new comprehensive stroke unit. **(A)** Patients with acute ischemic stroke are admitted to the stroke unit. Patients who receive neurosurgical procedures or have intracerebral or subarachnoid hemorrhage are admitted to the neurosurgical ICU. Depending on their trauma severity, patients with traumatic brain injury may be admitted to the trauma ICU or neurosurgical ICU. Patients with other life-threatening neurologic diseases, including status epilepticus, brain injury after cardiac arrest, cerebral edema from acute liver failure or central nervous system infection, and neuromuscular diseases with respiratory failure are admitted to the medical ICU. **(B)** More patients with intracerebral and subarachnoid hemorrhage will be admitted to the comprehensive stroke unit when it starts to operate.

Neurocritical illnesses account for 20–25% of patients requiring intensive care unit (ICU) in a developed country (6). Data on the burden of neurocritical diseases in Thailand is lacking, but it is thought to be higher in developing countries than in developed countries (4). In our institution, the specialized neurological ICU is unavailable. Neurocritical care patients are treated at various places operated by different departments (Figure 1A); this causes difficulty in monitoring and evaluating the efficacy of the treatment process and outcomes for these patients.

The benefits of specialized ICUs for neurocritical care patients are evident. Dedicated NICUs with neurointensivist-led teams have been shown to improve functional outcomes and reduce mortality, hospital length of stay, and treatment costs among critically-ill neurological patients (7–10). These benefits are demonstrated across various neurologic diseases, including strokes and traumatic brain injury (11–13). However, the shortage of ICU beds and intensive care nurses is the main problem in our country. Siriraj hospital is one of Thailand's largest academic and tertiary care hospitals. There are ~2,000 beds for inpatient care, but only 6% of the hospital beds are ICU beds, which are insufficient to accept all critically ill patients to the ICU^1^. Thus, in our circumstance, integrating neurocritical care into the current hospital workflow by collaboration between intensivists, neurologists, neurosurgeons, and other multidisciplinary care teams to take care of critically-ill neurologic patients at prevailing locations is more suitable than developing another specialized ICU because building a new NICU is costly, and needs new hospital staff recruitment and reorganization of the hospital system. As suggested in a previous article, the standard recommendations for the organization of NICUs are not feasible in resource-limited settings, and a multidisciplinary approach may be an acceptable alternative strategy to deliver neurocritical care to low- and middle-income countries (2).

Recently, led by vascular neurologists, our hospital has developed a new comprehensive stroke unit with 12-ICU beds that increase the capacity to take care of all types of acute stroke patients, acute ischemic stroke (AIS) and intracerebral (ICH) and subarachnoid hemorrhage (SAH). When it starts to operate, we expect more patients with ICH and SAH and patients with AIS who require critical care management will be admitted to this place. As a result, most patients with neurocritical illnesses, except traumatic brain and spinal cord injury and post-operative neurosurgical patients, will be treated in the stroke unit and medical ICUs (Figure 1B). Therefore, implementing neurocritical care in both places should be a good start from our standpoint.

## Practical implementation of neurocritical care in Thailand

Caring for patients with life-threatening neurologic conditions requires adherence to standard guidelines for the specific diseases causing primary brain injury and strategies to prevent, early detection, and prompt treatment of secondary brain injury resulting from intracranial hypertension, seizures, or systemic complications such as hypotension or hypoxemia (14). Proper management of all these complex conditions by neurointensivists can prevent further brain damage and improve patient outcomes (Table 1).

**Table 1:** Practical implementation of neurocritical care for the common neurologic diseases.

**Location**	**Disease**	**Critical care management of neurologic conditions**	**Critical care management of medical conditions**
Comprehensive stroke unit	Acute ischemic stroke	- Reperfusion therapy in eligible patients - Etiology identification - Blood pressure control - Monitoring for angioedema, hemorrhagic transformation, cerebral edema, and seizures	- Hemodynamic and respiratory stabilization - Maintenance of euvolemic status - Treatment of metabolic disturbances - Management of cardiopulmonary and infectious complications - Venous thromboembolism prophylaxis - Blood glucose and fever control - Dysphagia screening - Rehabilitation
	Intracerebral hemorrhage	- Blood pressure control - Coagulopathy reversal - Etiology identification - Treatment of vascular abnormalities - Treatment of intracranial hypertension - Monitoring for seizures	
	Subarachnoid hemorrhage	- Treatment of ruptured aneurysm - Treatment of intracranial hypertension - Administration of nimodipine - Monitoring for delayed cerebral ischemia - Monitoring for seizures	
Medical intensive care unit	Brain injury after cardiac arrest	- Definite treatment of specific causes - Targeted temperature management - Standard neuroprognostication	
	Status epilepticus	- Early seizure control - Electroencephalography monitoring - Etiology identification - Definite treatment of specific causes	
	Cerebral edema in acute liver failure	- Liver transplantation - Continuous renal replacement therapy - Treatment of intracranial hypertension	

In our hospital settings, eight intensivists, one with neurocritical care training, 14 neurologists, and 11 neurosurgeons are available 24 h a day, 7 days a week (15, 16). Four electroencephalography (EEG) machines are sufficient for the patients in the medical ICUs, but only one EEG technician is working for our hospital. Thus, 24-h continuous EEG monitoring is generally initiated during the workday. Computed tomography (CT) of the brain can be performed anytime, but magnetic resonance imaging (MRI) machines are limited.

To achieve effective implementation, we adapted the proposed strategies in pediatric neurocritical care: planning, educating, restructuring, financing, and managing quality (17). We focus on planning, educating, and managing quality and try to minimize changes in the hospital structure and financing system. The intensivist with neurocritical care training will lead the neurocritical care consultation service, education, and research.

### Planning

Our plan to incorporate neurocritical care into the hospital system includes neurocritical care consultation in the stroke unit and medical ICUs, neurocritical care education for internal medicine and neurology residents and critical care fellows, and research to understand the local common disease burden and treatment process.

### Neurocritical care consultation in the stroke unit

Optimal stroke care by a specialized interdisciplinary team including vascular neurologists, specialized nurses and rehabilitation professionals in the stroke unit has improved outcomes for acute stroke patients (18). In our hospital, most patients with AIS are treated in the stroke unit. However, patients with ICH and SAH are previously managed in other places; organized care in the stroke unit will also benefit these patients (19).

As shown in Figure 1B, the new structure, all patients with AIS are still treated in the stroke unit, but the patients with ICH or SAH may be admitted to the stroke unit or neurosurgical department depending on the discussion between vascular neurologists and neurosurgeons. Vascular neurologists are attending physicians at the stroke unit, and an intensivist works as a consultant for critical patients. The intensivist will systematically evaluate the patients and provide opinions on critical care management; the final plans will be carried out in agreement with the attending neurologist.

#### Acute ischemic stroke

Patients with AIS in our hospital are managed according to the current standard guidelines; intravenous thrombolysis and mechanical thrombectomy are available reperfusion strategies (20). Besides the definite therapy, ~one-fourth of the AIS patients need critical care management, and intensivists can fulfill this requirement (21).

Hemodynamic and respiratory management is essential issues for early AIS management. Blood pressure reduction is an important measure to prevent hemorrhagic transformation, but relative hypotension may cause inadequate cerebral perfusion and worsen neurologic symptoms (22). Patients who are comatose or have severe angioedema after intravenous thrombolysis need endotracheal intubation and appropriate mechanical ventilation management. Life-threatening cerebral edema requiring hyperosmolar therapy and surgical decompression can develop in patients with large hemispheric infarction due to middle cerebral artery occlusion or cerebellar infarction (23, 24). Symptomatic intracranial hemorrhage may occur after thrombolytic therapy and require immediate attention for coagulopathy reversal (20).

#### Intracerebral and subarachnoid hemorrhage

Caring for patients with ICH and SAH in the stroke unit is a novel workflow in our institution. The American Heart Association/American Stroke Association and Neurocritical Care Society guidelines are applied to manage these patients. Critical care management for ICH and SAH is complex. All these patients need stabilization of the airway, breathing, and circulation. Early ICH management focuses on limiting hematoma expansion by blood pressure control and reversal of coagulopathy, identifying specific causes, and evaluating for elevated intracranial pressure (25). Management of SAH focuses on the prevention of rebleeding by early treatment for ruptured aneurysm and blood pressure control, management of elevated intracranial pressure from cerebral edema, intraventricular hemorrhage and hydrocephalus, and detection and treatment of delayed cerebral ischemia by clinical examination and transcranial doppler ultrasonography (TCD) (26, 27). Endovascular and surgical interventions for cerebral aneurysms and arteriovenous malformation are available in our institution by radiointerventionists and neurosurgeons.

#### Systemic complications in acute strokes

Holistic care by intensivists to detect and timely manage neurologic and systemic medical complications can limit secondary brain injury. Seizures, hydrocephalus, and intracranial hypertension are common neurologic complications. Acute myocardial infarction, cardiac arrhythmias, stress cardiomyopathy, and hypoxemia from airway obstruction, aspiration, pneumonia, pulmonary embolism and neurogenic pulmonary edema are common cardiopulmonary complications in critical stroke patients (27–29). Venous thromboembolism (VTE) is a preventable complication in neurocritical care; the ICU-VTE score can be adopted to determine patients at high risk for VTE (30). Mechanical or pharmacologic thromboprophylaxis should be provided as appropriate, and early removal of central venous catheter and mobilization may further reduce the VTE risk (31). Besides, maintenance of euvolemic status, blood glucose and fever control, and treatment of hyponatremia are also important issues for these patients (25–28).

### Neurocritical care management in the medical intensive care unit

Post-cardiac arrest brain injury and status epilepticus are the most common neurologic conditions in our medical ICU. Recently, more patients with acute liver failure have been transferred from outside hospitals to our unit while waiting for liver transplantation. Central nervous system infections and neuromuscular diseases are infrequent. All these conditions require collaboration between attending intensivists and neurologists. Intensivists in different subspecialties work as attending physicians in the medical ICUs; one with neurocritical care training plays a role as an attending physician and consultant for critically-ill neurologic patients.

#### Brain injury after cardiac arrest

Brain injury is the primary cause of death and disability among cardiac arrest survivors (32). Multisystem management to minimize secondary brain injury includes definite treatment to prevent recurrent cardiac arrest episodes, restoration of systemic perfusion, prevention of hypoxemia and hyperoxia, optimal ventilation to achieve eucapnia, and active temperature control in comatose patients (33, 34). For accurate temperature control, we used esophageal probes for continuous core temperature monitoring and surface cooling devices with a temperature feedback system to control body temperature according to the standard guidelines until 72 h after the return of spontaneous circulation (35, 36).

Standardized neuroprognostication is essential to determine the high likelihood of poor functional outcomes in comatose patients without sedative medication or metabolic disturbances. Because of an uncertain threshold of neuron-specific enolase and the unavailability of the somatosensory evoked potential device, we used clinical examination, electroencephalography (EEG), and brain CT to assess neurological prognosis. The presence of at least two predictors according to the European Resuscitation Council and European Society of Intensive Care recommendations is used to classify patients as poor outcomes (33).

#### Status epilepticus

Generalized convulsive status epilepticus (GCSE) is another common neurologic condition requiring medical ICU admission. Since non-compliance to standard guidelines was associated with a longer duration of seizures and poor outcomes (37), developing a local treatment protocol that complies with standard guidelines is crucial to assure guideline adherence, especially appropriate doses and time of benzodiazepines and second-line antiseizure medications, and airway management (38, 39). Continuous EEG (cEEG) is increasingly used in our institution with coordination between intensivists and neurologists/epileptologists to confirm seizure suppression in patients who did not gain consciousness after antiseizure treatment and to guide dose adjustment of anesthetic drugs in patients with refractory status epilepticus (38). Moreover, cEEG helps diagnose psychogenic non-epileptic attacks (PNEA). Differentiating PNEA from GCSE among patients with prolonged convulsive activity is important because some PNEA patients received endotracheal intubation due to respiratory depression from high-dose benzodiazepine (40, 41).

#### Management of cerebral edema

Cerebral edema and intracranial hypertension can occur in both stroke and medical ICUs. Since an intracranial pressure (ICP) monitor has never been used in our hospital, the imaging and clinical examination (ICE) protocol and the Neurocritical care society's guidelines are applied (42, 43). Nonetheless, the ICP monitor is still helpful as it allows optimization of cerebral perfusion pressure (44). ICP monitoring can be used in patients with external ventricular drain placement in coordination with neurosurgeons.

In the medical ICU, acute liver failure (ALF) is the most common cause of cerebral edema, and intracranial hypertension is the leading cause of death in these patients (45, 46). Since cytotoxic brain edema from hyperammonemia is the predominant pathophysiology, continuous renal replacement therapy (CRRT), together with other strategies to reduce ICP, is initiated in all ALF patients with brain edema in coordination with critical care nephrologists (46–48). Other liver support therapy and plasmapheresis may also be used in appropriate conditions while waiting for liver transplantation. Control of serum sodium levels is also essential in this case. Raising serum sodium levels can be achieved by either administering hypertonic saline or adjusting fluid replacement in the CRRT; thus, communication between intensivists and nephrologists is necessary (49).

### Development of hospital-based protocols to guide the management of neurologic emergencies

We have recently developed our hospital-based protocols for status epilepticus, post-cardiac arrest care and neuroprognostication for comatose patients after cardiac arrest in collaboration between intensivists and neurologists based on the current evidence and standard guideline recommendations and our available resources, whereas the local protocol for revascularization of AIS patients created by vascular neurologists and neurointerventionists has been used for many years and regularly updated. International guidelines are applied with the best available resources for other neurocritical diseases.

### Education

Internal medicine and neurology residents and critical care fellows are involved in patient care and require education in critical care neurology. Management of neurocritical illnesses is integrated into the critical care curriculum for the critical care fellowship program.

We provide knowledge on common neurological problems in ICUs, including post-cardiac arrest care and standard neuroprognostication, intracerebral hemorrhage, cerebral edema and intracranial hypertension, status epilepticus, neuromonitoring, and appropriate use of analgesia and sedation to internal medicine and neurology residents and critical care fellows. Learning activities include classroom lectures, small-group interactive lectures, and active learning by presenting topic reviews. The intensivist with neurocritical care training is responsible for the teaching classes. We also have a weekly critical care grand round and conference where all challenging cases in the ICU including critically-ill neurologic patients are discussed between trainees and ICU staff in the different subspecialties.

### Quality management and research

Quality management and research are essential for effective implementation strategies (17). We will adopt the performance measure set proposed by the Neurocritical care society, consisting of twenty-one evidence-based performance measures specific to patients with neurocritical illnesses (50). Our plan begins with the performance measures for the most common neurocritical conditions in the stroke unit and medical ICUs, including baseline severity scale, admission unit, vascular imaging, acute interventions, symptomatic ICH after intervention and decompressive craniectomy in AIS, coagulopathy reversal and avoidance of corticosteroids in ICH, nimodipine administration and screening for vasospasm in SAH, appropriate dose and timing of benzodiazepines and antiseizure medications in status epilepticus, targeted temperature management in post-cardiac arrest, and venous thromboembolism prophylaxis.

For the research, we start with observational studies to understand the burden and impact of common neurocritical illnesses on patient outcomes and evaluate the performance of the treatment process. More innovative research will be possible when all those issues are addressed. Non-invasive continuous cerebral autoregulation monitoring using near-infrared spectroscopy to individualize patient blood pressure is one of our interests (51).

## Conclusion

Integrating neurocritical care into the available resource through the collaboration of multidisciplinary teams is a possible alternative strategy to deliver a standard of care to critically ill neurologic patients in resource-limited settings. The standard guidelines should be complied with and adjusted for appropriate resources. Providing neurocritical care education and developing research systems will lead to sustainable quality improvement in patient care.

## Author contributions

TV performed literature reviews and drafted and revised this manuscript.

## Conflict of interest

The author declares that the research was conducted in the absence of any commercial or financial relationships that could be construed as a potential conflict of interest.

## Publisher's note

All claims expressed in this article are solely those of the authors and do not necessarily represent those of their affiliated organizations, or those of the publisher, the editors and the reviewers. Any product that may be evaluated in this article, or claim that may be made by its manufacturer, is not guaranteed or endorsed by the publisher.

## Footnotes

^1^Available online at: https://www.si.mahidol.ac.th/annualreport/2018/PDF/20.pdf.
